# Molecular Epidemiology of Human Papillomaviruses, *Neisseria gonorrhoeae, Chlamydia trachomatis* and *Mycoplasma genitalium* among Female Sex Workers in Burkina Faso: Prevalence, Coinfections and Drug Resistance Genes

**DOI:** 10.3390/tropicalmed6020090

**Published:** 2021-05-27

**Authors:** Sessi Frida Tovo, Théodora Mahoukèdè Zohoncon, Amana Metuor Dabiré, Régine Ilboudo, Rahimatou Yasmine Tiemtoré, Dorcas Obiri-Yeboah, Albert Théophane Yonli, Essi Etonam Dovo, Rogomenoma Alice Ouédraogo, Abdoul Karim Ouattara, Pegdwende Abel Sorgho, Djénéba Ouermi, Florencia Wendkuuni Djigma, Charlemagne Ouédraogo, Lassana Sangaré, Jacques Simpore

**Affiliations:** 1 Laboratory of Molecular Biology and Molecular Genetics (LABIOGENE), Ouagadougou BP 7021, Burkina Faso; fridaslc@yahoo.fr (S.F.T.); zohoncont1@yahoo.fr (T.M.Z.); ametuordabire@yahoo.fr (A.M.D.); thiombianomina@yahoo.fr (R.Y.T.); yonlitheo@yahoo.fr (A.T.Y.); lauress12@gmail.com (E.E.D.); oouedraogoralice@yahoo.com (R.A.O.); ak_ouattara@labiogene.org (A.K.O.); abelcyriac.sorgho@yahoo.fr (P.A.S.); oddianemed@gmail.com (D.O.); flodjig@yahoo.fr (F.W.D.); 2 Biomolecular Research Center Pietro Annigoni (CERBA), Ouagadougou BP 364, Burkina Faso; regineilboudo586@yahoo.com (R.I.); ocharlemagne@yahoo.fr (C.O.); 3 Saint Camille Hospital of Ouagadougou (HOSCO), Ouagadougou BP 444, Burkina Faso; 4 Faculty of Health Sciences, University Saint Thomas d’Aquin (USTA), Ouagadougou BP 10212, Burkina Faso; 5 Department of Biochemistry and Microbiology, University of Dédougou, Dédougou BP 176, Burkina Faso; 6 Department of Microbiology and Immunology, School of Medical Sciences, University of Cape Coast, Cape Coast P.O. Box 5007, Ghana; castella.oy@gmail.com; 7 Department of Bacteriology-Virology, University Joseph KI-ZERBO, Ouagadougou BP 7021, Burkina Faso; sangarelassana01@gmail.com; 8 Laboratory of Bacteriology and Virology, Yalgado Ouédraogo University Hospital, Ouagadougou BP 5234, Burkina Faso

**Keywords:** HPV, *Neisseriae gonorrhoeae*, STIs, PCR, sex workers, resistance genes

## Abstract

Viral and bacterial infections represent an occupational risk for female sex workers. This study aimed at determining HPV coinfection with genital pathogens among female sex workers in West and Central Africa and identifying antibiotic resistance genes. A total of 182 samples from female sex workers were analyzed by real-time PCR and classic PCR. For the molecular diagnosis of HPV, the real-time multiplex amplification kit “HPV Genotypes 14 Real-TM Quant” from SACACE Biotechnologies^®^, detecting 14 high-risk HPV genotypes, was used, while for other pathogens, the real-time multiplex amplification kit *N. gonorrhoeae*/*C. trachomatis*/*M. genitalium*/*T. vaginalis* Real-TM, allowing their simultaneous detection, was used. The women were aged 17–50 years with an average age of 27.12 ± 6.09 years. The pathogens identified were HPV 54.94% (100/120), *Neisseria gonorrhoeae* (13.74%), *Chlamydia trachomatis* (11.54%) and *Mycoplasma genitalium* (11.54%). The most common HPV genotypes were HPV68, HPV38 and HPV52. The antibiotic resistance genes identified were *bla* _QNR B_ 24.00%, *bla* _GES_ 22.00%, *bla* _SHV_ 17.00%, *bla*_CTX-M_ 13.00% and *bla* _QNR S_ 1.00%. This study revealed the presence of various HPV genotypes associated with other pathogens with problems of antibiotic resistance among sex workers of West and Central African origin working in Ouagadougou.

## 1. Introduction

Genital infections represent a great public health concern. They can facilitate infection with HIV, HPV and many other sexually transmitted infections (STIs) such as *Neisseria gonorrhoeae* (*NG*), *Chlamydia trachomatis* (*CT*), *Mycoplasma genitalium* (*MG*) and *Trichomonas vaginalis* (*TV*) [1]. STIs are serious public health concerns around the world because of their frequency and associated complications [1], with *Chlamydia trachomatis* often being the most common. *Neisseria gonorrhoeae* also remains a pathogenic bacterium of considerable health risk, especially in developing countries [2,3,4,5]. It should be noted that many forms are asymptomatic; more than 60% of female urogenital infections and more than 90% of pharyngeal infections are asymptomatic [6]. The three bacterial species—*NG*, *CT*, and *MG*—are able to cross the cervical barrier and infect glandular crypts, therefore leading to endocervicitis. In addition, HPV infection is most prevalent in a risk population for sexually transmitted infections (STIs). To this effect, the objectives of the study were to characterize by real-time PCR the pathogens such as *NG*, *CT*, *MG* and *TV* responsible for STIs in female sex workers in West and Central Africa with known HPV status so as to detect antibiotics resistance genes.

## 2. Materials and Methods

### 2.1. Setting of the Survey

The study was conducted in Ouagadougou, both in the Gynecology Department of Saint Camille Hospital in Ouagadougou and the Molecular Biology Laboratory of the Pietro Annigoni Center for Biomolecular Research (CERBA) in Burkina Faso. It was a cross-sectional study with descriptive and analytical aims, which took place from July 2017 to July 2020.

### 2.2. Study Variables

The following variables were studied: sociodemographic characteristics (age, nationality, level of education, marital status); HIV status; HPV molecular diagnostic results by real-time PCR; and pathogen status (*N. gonorrhoeae*, *C. trachomatis*, *M. genitalium*, *T. vaginalis*).

### 2.3. Sampling

A first awareness campaign was conducted for a month to identify the sex workers. The goal was to raise their awareness and invite them to receive screening for cervical cancer. Female sex workers were included in the study after their consent was obtained. After their identification and after their written informed consent was obtained, the second phase consisted of sample collection. The sample size (182) was obtained following a 4-week collection. Each patient was placed in a gynecological position on the examination table. After the cervix and anogenital areas were highlighted using a disposable speculum, a cotton swab for specimen collection was inserted into the endocervix and ectocervix at the squamocolumnar junction. The collected specimen was placed in a transport medium (SACACE Amplification Kit Transport Medium) and stored at −20 °C pending DNA extraction.

This study consisted of analyzing data from 182 female sex workers who were living in Burkina Faso at that period. They were from nine (09) nationalities namely: Burkina Faso, Nigeria, Cameroon, Benin, Togo, Senegal, Ghana, Côte d’Ivoire and Mali.

An endocervical cervical sampling was performed for them in 2017; these included all women who met the recommended conditions for good vaginal sampling (VSP), and whose samples after HPV testing were properly stored at minus eighty degrees Celsius (−80 °C) for further molecular analyses, including those in this study.

### 2.4. Molecular Characterization

#### 2.4.1. DNA Extraction

DNA extraction was performed at CERBA/LABIOGENE using the DNA-Sorb-A kit from SACACE Biotechnologies^®^ (Reference K-1-1/A/100, Lot 23B16A155), following the protocol provided by the manufacturer. For the molecular diagnosis of HPV, the “HPV Genotypes 14 Real-TM Quant” real-time amplification kit from SACACE Biotechnologies^®^ (Reference V67-100FRT, Lot 26 G17K414) detecting 14 high-risk HPV genotypes was used (HPV 16, 18, 31, 33, 35, 39, 45, 51, 52, 56, 58, 59, 66.68). PCR was performed using the SaCycler-96 real-time PCR device in version 7.3 of SACACE Biotechnologies^®^. For other pathogenic germs, the Sacace Biotechnologies *N. gonorrhoeae*/*C. trachomatis*/*M. genitalium*/*T. vaginais* Real-TM analysis protocol kit was used, following the protocol provided by the manufacturer and allowing their simultaneous detection.

#### 2.4.2. Real-Time PCR Amplification for the Urogenital STI Pathogens

The real-time PCR performed was multiplexed with four tubes for each sample. The reaction mixture yielded a volume of 25 µL, i.e., 10 µL of the volume of DNA and 15 µL of the preparation of PCR mix-1-FL, PCR mix-2-FRT and polymerase (TaqF) (10 µL of PCR mix-1-FL, 5 µL of PCR mix-2-FRT and 0.5 µL of polymerase (TaqF). The mixture was vortexed and distributed to each well. The program used is the Sacace™ for the urogenital “STD” assays proposed by the manufacturer. This amplification program was: 1 cycle of 95 °C for 10 min followed by 5 cycles of 95 °C for 5 s; 60 °C for 20 s; 72 °C for 15 s and finally 40 cycles of 95 °C for 5 s; 60 °C for 30 s and 72 °C for 15 s. An internal amplification control and negative and positive controls were used. Interpretation of the results was performed using the Microsoft Excel Starter 2010, Real-Time Program provided by the manufacturer (Microsoft, Redmond, WA, USA).

#### 2.4.3. Identification of Resistance Genes by Classical PCR

In order to detect antibiotic resistance genes, specific primers were used (Table 1). The PCR was performed in a 20.00 µL reaction volume consisting of master mix 5× HOT FIREPol@ Blend, 4.00 µL; primer F, 0.60 µL; primer R, 0.60 µL; H_2_O, 10.80 µL; and DNA, 5.00 µL. For the amplification of the desired genes, several programs were used. Antibiotic resistance genes were detected by amplification using the Gene Amp System PCR 9700 thermal cycler (Applied Biosystems, Foster City, CA, USA). Migration was performed by electrophoresis on a 2.00% agarose gel at 120 volts for 40 min. Visualization was performed using ethidium bromide (BET). A 100 bp molecular weight marker was used as a molecular weight index.

#### 2.4.4. Data Analyses

Data were entered and analyzed and tables and figures were produced using the IBM SPSS software version 21 and Epi Info 6 and Microsoft Excel 2007. The Chi-square test was used for comparisons with a significant difference for *p* ˂ 0.05.

## 3. Results

Our study population was composed of 182 women aged 17 to 50 years with an average age of 27.12 ± 6.09 years. Women with an age less than or equal to 30 years were the most infected by pathogens *NG*, *CT*, *MG* and HPV. Concerning the HIV status of female sex workers, 7/182 were HIV positive or 3.85%. The secondary and university schooling levels were respectively 35.71% and 20.33% by HPV and pathogens (*NG*, *CT*, *MG*) identified (Table 2).

Table 3 indicates the pathogens *NG*, *CT*, *MG* encountered in our study.

In our study population, 56 women out of 182 (or 30.77%) were infected with at least one of the pathogens encountered. For bacteria, 13.74% of the study population were infected with *NG*, 11.53% were infected with *CT* and 11.53% were infected with *MG*. *Trichomonas vaginalis* was not found in this study. Coinfection *NG*/*MG*/*CT* was encountered in 5.49% of patients. Of these, 3.30% of patients were coinfected with *CT* and *MG*, 1.65% of patients were coinfected with *NG* and *CT*, and 0.55% of patients were infected with *NG, CT* and *MG*. However, HPV/*CT*/*MG*/*NG* coinfection was found in 30.00% of female sex workers. There is no statistically significant difference between HPV/*NG* and HPV/*NG*/*MG* coinfection.

**Figure 1:**
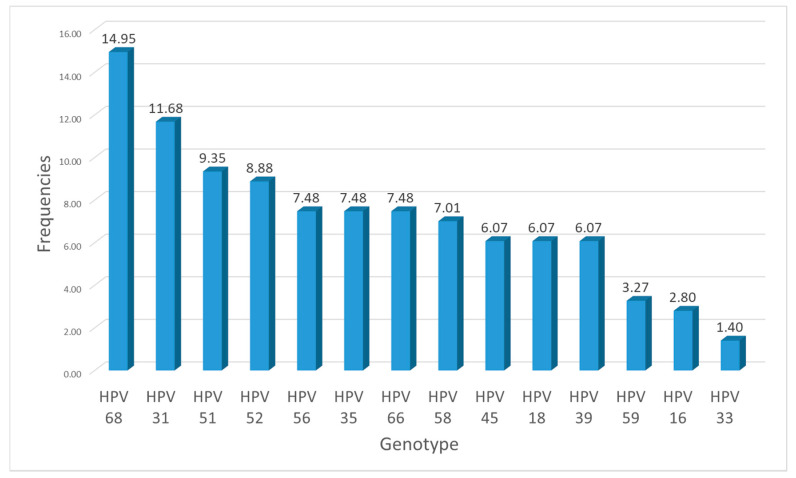
Histogram of the 14 HPV genotypes found in our study in relation to their total number.

All 14 HPV genotypes were found (Figure 1). The HPV 16 and/or HPV 18 genotypes were among the combinations found in the coinfection of HPV with the other pathogens. *N. gonorrhea* was associated with five combinations of the HPV genotype, such as HPV31, HPV45, HPV59, HPV58 and HPV52, while *C. trachomatis* was associated with four genotype combinations (HPV16, HPV31, HPV68 and HPV52 and HPV16, HPV59, HPV35 and HPV52).

### HIV Status of Patients

Negative HIV status was reported by female sex workers in 92.86% of cases, while 3.85% of female sex workers reported HIV positive status. In addition, 3.29% had an unknown serological status. Coinfection with HIV and HPV (HPV68, HPV52 and HPV51; HPV 31; HPV 18; HPV 31, HPV52, HPV68 and HPV56) was found in 14.28% of cases.

It is worth noting that beyond the HIV/HPV coinfection, there was also the presence of two bacteria namely *NG* and *CT*.

**Figure 2:**
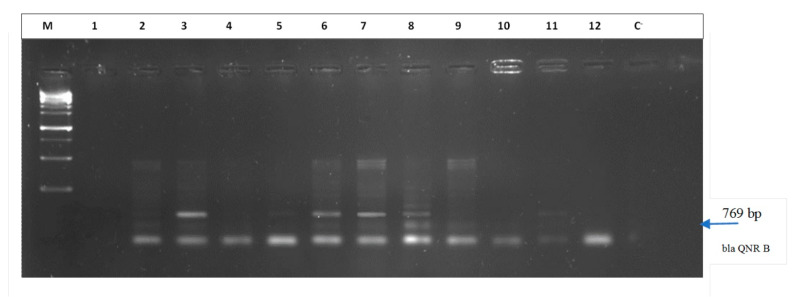
Agarose gel image showing the PCR products of the *bla* _QNR B_ gene. Lane (M) = molecular weight marker; Lane (3; 6; 7; 8; 11) = positive to *bla* _QNR B_ gene; Lane (C^−^) = negative control.

The presence of the *bla*_QNB_ gene is shown in Figure 2.

**Figure 3:**
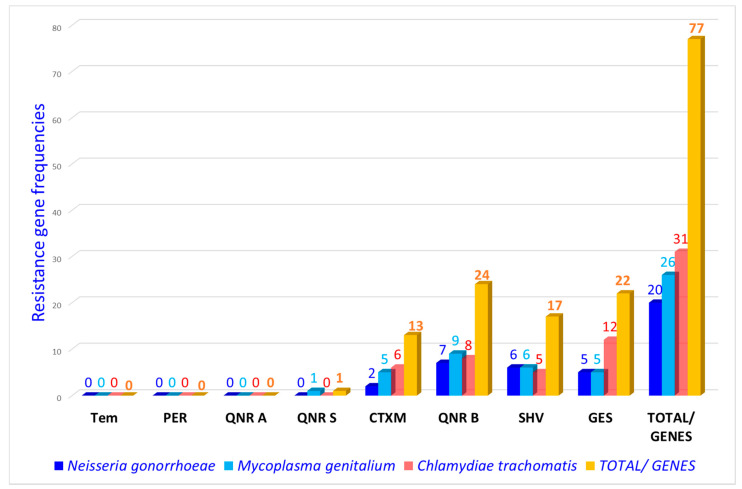
Antibiotics resistance genes as a function of bacteria.

A total of eight (08) resistance genes were sought, of which five (5) were tested positive (Figure 3). These are *bla* _CTX-M_ 13.00%, *bla*_SHV_ 17.00%, *bla* _QNR B_ 24.00%, *bla* _QNR S_ 1.00%, and *bla* _GES_ 22.00%. Of the bacterial species studied, *M. genitalium* was the only species to simultaneously host the five resistance genes found: *bla* _CTX-M_, *bla* _HVS_, *bla* _GES_, *bla* _QNR B_ and *bla* _QNR S_. The percentage of antibiotic resistance genes found was 77.00% with a predominance of *bla* _QNR B_ (Figure 3). 

Table 4 shows coinfection with HPV and *NG, CT, MG, TV*. There is no statistically significant difference between HPV/*NG* and HPV/*NG*/*MG* coinfections. This high rate of coinfection has the combinations HPV/*NG*, HPV/*CT*, HPV/*MG*, HPV/*NG*/*CT*, HPV/*NG*/*MG* and HPV/*CT*/*MG* with no statistically significant difference for HPV/*NG* and HPV/*NG*/*MG*.

## 4. Discussion

Among HPV-infected women, according to this study, the age range of 21–30 years was the most represented, which is also the most indicated age range for female sex workers infected with MG/NG/CT pathogens. According to several authors, the age range of 21–30 years is the period when women are most exposed to HPV infection and other STIs [7,8]. This is also the age when young women are physically fit, in good physical shape and more sexually active [7,8]. This high level of sexual activity would partly explain the high prevalence of HPV and STIs in these sex workers.

Detecting viral and bacterial coinfection is a critical issue in a population at risk such as sex workers. The present study has made it possible to highlight this HPV/*NG*/*MG*/*CT* coinfection among sex workers in Burkina Faso.

Most of the countries concerned by our study share their borders with Burkina Faso, which therefore facilitated women’s easy access to the country. They migrated into Ouagadougou because they found easy access. In addition, the other reason is that they did not want to be recognized as sex workers in their home countries [9,10]. In terms of HIV status, most of the patients in this group of female sex workers had negative HIV status. While six had unknown HIV status, seven were HIV positive. There is a relatively low prevalence of HIV-positive status; however, several studies have shown that genital infections and STIs can increase HIV infectivity and/or transmission during sexual intercourse [11,12]. This low rate of positive HIV status may be explained by the fact that in most cases, sex with female sex workers is protected or patients may not have answered truthfully to the question about their HIV status [11,12]. The association of STIs among sex workers in this investigation would increase the risk for their sexual partners. Indeed, although genital infections are often asymptomatic in women, the presence of a low genital infection would increase the risk of contracting HPV. For example, bacterial vaginosis is believed to be associated with high levels of anaerobic organisms that can damage the vaginal epithelium and thus increase the risk of HPV infection. In addition, Coudray and Madhivanan reported that bacterial vaginosis may increase the risk of contracting many STIs such as HIV, *NG*, *CT*, *TV*, and herpes simplex-2 virus (HSV-2) [13]. In addition, female sex workers constitute a “reservoir” of STIs, which would further increase the risk of genital infection for the general population. HPV coinfection with pathogens such as *NG*, *CT*, *MG* and *TV* have been reported by other authors such as Coudray and Madhivanan in 2020 in the USA and Lv et al. 2019 in China [14,15]. HPV is the leading cause of cervical cancer, which is the leading cause of cancer death in women in Africa. It is therefore a real public health problem, especially because some of the circulating genotypes are not covered by existing and available vaccines [16,17,18]. Pathogens such as *NG*, *MG* and *CT* would promote HPV infection which in turn could persist and induce precancerous lesions or cervical intraepithelial neoplasia type 1, 2 or 3 (CIN1, CIN2 and CIN3), which may regress or progress to cervical cancer.

Our study on HPV in female sex workers found 54.94% (100/182) of HPV-positive cases. Several authors have (unlike us) conducted studies in the general female population, particularly in sexually active women. According to the study by Zohoncon et al. in 2020 on HPV in the general population of five West African countries, there is a prevalence of (717/2133) 33.61% of high-risk HPV infection (Tovo vs. Zohoncon, *p* < 0.001). Traoré et al. (2016) in Burkina Faso, in their study on HPV among general population women who are not sex workers, found a prevalence of (46/120) 38.3% (Tovo vs. Traore, *p* < 0.005). HPV infection is higher in women who are sex workers than in the general population [7,17].

Thus, the treatment of these pathogens detected would make it possible to break the chain of transmission of these genital infections for the health of populations.

With regard to resistance genes, our study revealed the presence of several genes hosted by the different species studied. These resistance genes were also identified in previous studies in Burkina Faso but in other biological samples such as urine, fecal matter and pus. In contrast to our study in which *bla* _QNR B_ is predominantly represented, the study by Dabiré et al. in 2013 showed a predominance of *bla*_CTX-M_. The coexistence of several resistance genes observed in the present study was also detected in previous studies in Burkina Faso [19,20,21,22]. This coexistence of several resistance genes within bacteria is believed to confer multidrug resistance to antibiotics.

## 5. Conclusions

Despite all the control measures that have been taken so far against STIs, this study showed that female sex workers of West and Central African origin were infected with *NG*, *MG*, *CT* and HPV. This confirms that the presence of STIs remains a real public health issue. The magnitude of STIs among female sex workers in West and Central Africa and the detection of associated resistance genes require increased surveillance of the molecular epidemiology of these pathogens.

## Figures and Tables

**Table 1:** Primers used and antibiotic resistance genes sizes.

TEM-F: 5′-ATAAAATTCTTGAAGACGAAA-3′ TEM-R: 5′-GACAGTTACCAATGCTTAATCA-3′	*bla* _TEM_	1000 Pb
CTX-MF: 5′-GTTACAATGTGTGAGAAGCAG-3′ CTX-M-R: 5′-CCGTTTCCGCTATTACAAAC-3′	*bla* _CTX-M_	1000 Pb
PER-F: 5′-ATGAATGTCATTATAAAAGC-3′ PER-R: 5′-AATTTGGGCTTAGGGCAGAA-3′	*bla* _PER_	925 Pb
QNR A-F: 5′-ATTTCTCACGCCAGGATTTG-3′ QNR A-R: 5′-GATCGGCAAAGGTTAGGTCA-3′	*bla* _QNR A_	846 Pb
QNR B-F: 5′-GATCGTGAAAGCCAGAAAGG-3′ QNR B-R: 5′-ACGATGCCTGGTAGTTGTCC-3′	*bla* _QNR B_	769 Pb
QNR S-F: 5′-ACGACATTCGTCAACTGCAA-3′ QNR S-R: 5′-TAAATTGGCACCCTGTAGGC-3′	*bla* _QNR S_	566 Pb
SHV-F: 5′-ATG-CGTTATATTCGCCTGTG-3′ SHV-R: 5′-TTAGCGTTGCCAGTGCTC-3′	*bla* _SHV_	875 Pb
GES-F: 5′-ATGCGCTTCATTCACGCAC-3′ GES-R: 5′-CTATTTGTCCGTGCTCAGG-3′	*bla* _GES_	863 Pb

**Table 2:** Sociodemographic characteristics of the study population presented by identified pathogens.

Sociodemographic Characteristics	Positive	Negative	*p* Value	Positive	Negative	*p* Value
	N = 56 (%)	N =126 (%)		N = 100 (%)	N = 82 (%)	
Age Classes						
˂21	3 (1.65)	16 (8.79)		13 (7.14)	6 (3.30)	
21–30	17 (9.34)	101 (55.49)		63 (34.62)	55 (30.22)	
>30	1 (0.55)	44 (24.18)	0.07	24 (13.18)	21 (11.54)	0.45
Scholastic Level						
Not attending school	7 (3.85)	29 (15.93)		16 (8.79)	20 (10.99)	
Primary	12 (6.60)	29 (15.93)	0.2	19 (10.44)	22 (12.09)	0.08
Secondary & University	37 (20.33)	68 (37.36)		65 (35.71)	40 (21.98)	
SEXUAL PARTNERS						
≤500	53 (29.12)	116 (63.73)	0.75	94 (51.65)	75 (41.21)	0.5
>500	3 (1.65)	10 (5.59)		6 (3.30)	7 (3.84)	
MARITAL SITUATION						
Unmarried	50 (27.47)	118 (64.83)	0.47	95 (52.20)	73 (40.11)	0.13
Married	6 (3.30)	8 (4.40)		5 (2.75)	9 (4.94)	
NATIONALITIES						
Burkinabè	19 (10.44)	60 (32.97)		42 (23.08)	37 (20.33)	
Nigerian	31 (17.03)	49 (26.92)	0.11	46 (25.28)	34 (18.68)	0.82
Others	6 (3.30)	17 (9.34)		12 (6.59)	11 (6.04)	
Totals	56 (30.76)	126 (69.23)	˂0.0001	100 (54.94)	82 (45.05)	NS

NS = X > 0.05.

**Table 3:** Frequency of pathogens identified by real-time PCR.

Pathogens Germs N = 182	Positive (%)	Negative (%)	*p*-Value
Only one Infection			
*MG*	21 (11.54)	161 (88.46)	
*CT*	21 (11.54)	161 (88.46)	0.76
*NG*	25 (13.74)	157 (86.26)	
*TV*	0.00	182 (100)	
Total (at least one infection)	56 (30.77)	126 (69.23)	
Coinfections			
*MG*/*CT*	6 (3.30)	176 (96.70)	
*MG*/*NG*	2 (1.10)	180 (98.90)	0.20
*CT*/*NG*	4 (2.20)	178 (97.80)	
*MG*/*NG*/*CT*	1 (0.55)	181 (99.45)	

**Table 4:** Coinfection with HPV, *NG*, *CT*, *MG* and *TV* among sex professionals in our study. Vaginal infections: cumulative NG/CT/MG/TV.

Coinfections	HPV+ N = 100	HPV− N = 82	*p* Value
*NG+*	15 (15.00%)	10 (12.19%)	0.74
*NG−*	85 (85.00%)	72 (87.80%)	
*CT+*	8 (8.00%)	13 (15.85%)	0.16
*CT−*	92 (92.00%)	69 (84.14%)	
*MG+*	12 (12.00%)	9 (11.00%)	0.83
*MG−*	88 (88.00%)	73 (89.00%)	
*NG+CT+*	2 (2.00%)	2 (2.43%)	0.76
*NG−CT−*	98 (98.00%)	80 (97.56%)	
*NG+MG+*	2 (2.00%)	0 (0.00%)	-
*NG−MG−*	98 (98.00%)	82 (100.00%)	
*CT+MG+*	2 (2.00%)	4 (4.87%)	0.51
*CT−MG−*	98 (98.00%)	78 (95.12%)	
Vaginal infections	30 (30.00%)	26 (31.71%)	0.80
No Vaginal infections	70 (70.00%)	56 (68.29%)	

## Data Availability

The datasets used and/or analyzed during the current study are available from the corresponding author on reasonable request.
